# Liver Bed Infiltration With Isobaric Levobupivacaine Versus Intravenous Paracetamol Analgesia for Postoperative Pain Management in Patients Undergoing Laparoscopic Cholecystectomy

**DOI:** 10.7759/cureus.76644

**Published:** 2024-12-30

**Authors:** Shilaga Dhar, Amandeep Kaur, Reecha Panghal, Mubadil Mushtaq, Ivneet Kaur, Aastha Gupta, Pankaj Kumar, Falak Deeba

**Affiliations:** 1 Anesthesia, Adesh Medical College and Hospital, Kurukshetra, IND; 2 Anesthesia, Ujala Healthcare Services Private Limited, Kashipur, IND; 3 Anesthesia, Mahavir Sansthan Cancer Hospital, Patna, IND; 4 Anesthesia, Rohilkhand Medical College and Hospital, Bareilly, IND

**Keywords:** cholecystectomy, levobupivacaine, outcomes, pain, satisfaction

## Abstract

Objectives

The study aimed to compare the use of isobaric levobupivacaine for liver bed infiltration and intravenous analgesia with paracetamol (acetaminophen) in managing postoperative pain in patients undergoing laparoscopic cholecystectomy.

Methods

An observational study was conducted on 80 patients scheduled for elective laparoscopic cholecystectomy. Inclusion criteria comprised adults of American Society of Anesthesiologists grade I, aged 18-65, undergoing elective surgeries. Exclusion criteria were contraindications to local anesthetics, history of chronic pain disorders, or ongoing opioid therapy. Participants were divided into two groups: Group LB receiving 30-40 ml 0.5% isobaric levobupivacaine for liver bed infiltration, and Group P receiving paracetamol 1 g intravenous during surgery just after removing the gallbladder out of the laparoscopic port. Primary outcomes included pain scores assessed using the visual analog scale (VAS) at one hour, six hours, 12 hours, 18 hours, and 24 hours postoperatively. Secondary outcomes included the total analgesic consumption, patient satisfaction scores, and any adverse effects experienced.

Results

In Group LB, the VAS scores showed a declining trend, starting at 6.4 ± 0.8 at one hour and decreasing to 4.5 ± 1.5 at six hours, 4.1 ± 1.7 at 12 hours, 2.6 ± 1.3 at 18 hours, and 1.3 ± 0.6 at 24 hours. In Group P, the VAS scores also decreased, beginning at 7.2 ± 1.1 at one hour, then dropping to 5.2 ± 1.2 at six hours, 4.8 ± 1.1 at 12 hours, 3.2 ± 0.9 at 18 hours, and 1.8 ± 0.5 at 24 hours. However, statistically, VAS scores were significantly lower in Group LB as compared to Group P at all time points: one hour, six hours, 12 hours, 18 hours, and 24 hours (p<0.05). Group LB demonstrated significantly better outcomes compared to Group P, including a longer time to first rescue analgesia (12 vs. five hours, p<0.0001), a reduced dose of additional analgesic requirement (3 ± 1 vs. 6 ± 2 µg/kg, p<0.0001), and higher patient satisfaction (38 (95%) vs. 23 (57.5%), p=0.0001). The incidence of nausea (3 (7.5%) vs. 6 (15%), P=0.481) and vomiting (5 (12.5%) vs. 8 (20%), p=0.363) was comparable between Group LB and Group P.

Conclusion

The use of levobupivacaine for liver bed infiltration provided adequate pain relief following laparoscopic cholecystectomy in comparison to paracetamol intravenous analgesia. Side effects like nausea and vomiting were statistically comparable in both groups. Overall, levobupivacaine for liver bed infiltration is a safe and effective option for controlling pain and increasing satisfaction among patients undergoing laparoscopic cholecystectomy.

## Introduction

Laparoscopic cholecystectomies are one of the most common abdominal surgeries performed worldwide [1-3]. For cholelithiasis, they remain one of the gold standard procedures. However, re-hospitalization after surgery may occur because of postoperative pain and overall increased patient dissatisfaction. Postoperative pain is one of the primary complaints in the recovery period after cholecystectomies [4].

Various local anesthetics, such as ropivacaine, bupivacaine, levobupivacaine, and lidocaine, are advised for controlling pain during laparoscopic cholecystectomy. Among these, bupivacaine is the most commonly used [5-8]. Like bupivacaine, its isomer levobupivacaine is long-acting and has a favorable side effects profile as compared to other alternatives, such as lidocaine and ropivacaine [2]. Therefore, levobupivacaine was chosen in the present study to compare against the common non-steroidal anti-inflammatory drug (NSAID), paracetamol.

Levobupivacaine is one of the common local anesthetic drugs used in laparoscopic cholecystectomies [7]. It controls pain by inhibiting the transmission of the pain by blocking sodium entry and binding to voltage-gated sodium channels [8]. On the other hand, paracetamol inhibits the synthesis of prostaglandins by inhibiting the cyclooxygenase enzyme [9,10]. 

Studies have continued to explore which of these two categories of drugs holds superiority over the other, as well as the effectiveness of various administration methods, such as nerve blocks, intraperitoneal injections, or injections at the site of incision method [6]. A previous study compared intraperitoneal bupivacaine against intravenous ketorolac and found that both bupivacaine and ketorolac were safe and effective in controlling pain [1]. One study showed that levobupivacaine and bupivacaine are equally effective in pain control in hernia surgeries [11]. Another study by El-Labban et al. [12] showed that the intra-incisional infiltration of levobupivacaine is more effective than the intraperitoneal route in controlling postoperative abdominal pain and decreasing the need for rescue analgesia. However, to date, no study has compared levobupivacaine for liver bed infiltration against intravenous paracetamol.

The present study was, thus, conducted to determine which of the two holds superiority over the other so that better postoperative pain control may be provided to patients undergoing laparoscopic cholecystectomy.

## Materials and methods

An observational study was conducted over a period of 18 months from July 2022 until December 2023. Institutional ethical clearance was obtained for the study. Criteria followed for inclusion were patients of American Society of Anesthesiologists (ASA) grade I, in the age group of 18-65, who underwent laparoscopic cholecystectomy at our hospital.

Any patient who had a history of chronic pain disorder, contraindication to local anesthetic, history of hospitalization within a month, hypersensitivity to drugs, heart diseases such as arrhythmias, cancers, and pregnancy were excluded from the present study. 

All laparoscopic cholecystectomy procedures were conducted by a single surgeon. The patients were alternately assigned to one of the two groups without randomization or blinding: (i) Group LB, which received 30-40 ml 0.5% isobaric levobupivacaine for liver bed infiltration; and (ii) Group P, which received intravenous paracetamol 1 g.

Sample size calculation

In the pilot study, the average pain scores (mean ± SD) in Group LB were 4.1 ± 1 at 12 hours and 1.2 ± 0.6 at 24 hours. For Group P, the scores were 4.8 ± 0.8 at 12 hours and 1.8 ± 1 at 24 hours. Based on these reference values, a minimum of 40 participants per group was needed to achieve a 90% study power and a 5% significance level. Therefore, the total sample size selected for the study was 80 participants, with 40 in each group.

Methodology

After taking written informed consent from each patient, the patients were explained each step of the study. The demographic details such as age, sex, body weight, physical status, and surgery duration were documented.

All patients underwent laparoscopic cholecystectomies under general anesthesia. Each patient was first administered 3-5 mL/kg of Ringer's lactate solution. Anesthesia induction was done by midazolam 20 µg/kg, fentanyl 2 µg/kg, and propofol 2 mg/kg. Atracurium 0.5 mg/kg was used to enable muscle relaxation for tracheal intubation. After a three-minute wait, intubation was performed with tubes ranging from 7.5-8 mm in diameter. Anesthesia was maintained with 60:40 oxygen:nitrous oxide (N_2_O) and isoflurane. During the operation, additional doses of fentanyl (1 µg/kg) and atracurium (0.2 mg/kg) were administered intravenously, as needed.

After completion of the surgery, once the gallbladder was out, levobupivacaine liver bed infiltration via epigastric port was given to Group LB, whereas intravenous paracetamol 1 g was given to Group P. Injection glycopyrrolate (10 mcg/kg) and neostigmine (0.05 mg/kg) were administered to reverse the muscle relaxation, following which the patients were extubated and taken out of the operation theatre and taken to the postanesthesia care unit (PACU). In the PACU, rescue analgesia was given to patients of both groups (as needed) in the form of intravenous fentanyl. For patients with a VAS score of 4 or above, a bolus dose of fentanyl (1 ug/kg) intravenous was administered, followed by a slow intravenous dose of 0.5 mg/kg meperidine, if necessary. For those with nausea or vomiting, 10 mg of metoclopramide was administered intravenously.

Outcome measures

Primary outcomes included pain scores assessed by VAS at one hour, six hours, 12 hours, 18 hours, and 24 hours postoperatively. The VAS rates pain from 0 to 10, where 0 signifies no pain, 1-3 represents mild pain that doesn’t interfere with sleep, 4-6 indicates moderate pain, 7-9 reflects severe pain that disrupts sleep, and 10 represents the most intense pain [13].

Secondary outcomes included total analgesic consumption, patient satisfaction scores, and any adverse effects (postoperative nausea and vomiting (PONV)). PONV scores, ranging from 0 to 4, captured the severity of nausea and vomiting, with 0 indicating no symptoms and 4 indicating very severe symptoms. Vomiting was defined as the forceful ejection of stomach contents or retching; episodes separated by more than one minute were counted as distinct events [1].

Patient satisfaction was rated 24 hours after surgery subjectively by the patients as 'satisfied' or 'not satisfied'.

Statistical analysis

Categorical variables were reported as frequencies and percentages. Quantitative data with normal distribution were presented as means ± SD, while non-normal data were expressed as medians with interquartile ranges. Data normality was tested using the Shapiro-Wilk test, and non-parametric tests were used for non-normal data. Mann-Whitney and independent t-tests compared quantitative variables, while the chi-square test was used for qualitative variables, with Fisher's exact test for cells with expected values <5. Data entry was done in Microsoft Excel (Microsoft Corp., Redmond, USA) and analysis was performed using IBM SPSS Statistics version 25.0 (IBM Corp., Armonk, USA). Statistical significance was set at p<0.05.

## Results

The demographic characteristics were statistically comparable between the two groups, as shown in Table 1.

**Table 1:** Patients’ baseline and intraoperative characteristics ASA PS: American Society of Anesthesiologists Physical Status; ^* ^Independent t-test; ^† ^Chi-square test; ^‡ ^Fisher's exact test

Parameters	Group LB (n=40)	Group P (n=40)	p value
Age (years)	38.65 ± 13.7	41.8 ± 9.5	0.228^*^
Gender (Male/Female)	7/33	10/30	0.412^†^
Weight (kg)	71.82 ± 10.4	75.3 ± 8.5	0.103^*^
ASA PS (I/II)	36/4	34/6	0.737^‡^
Operation time (minutes)	48.23 ± 11.4	51.8 ± 9.8	0.134^*^

**Figure 1:**
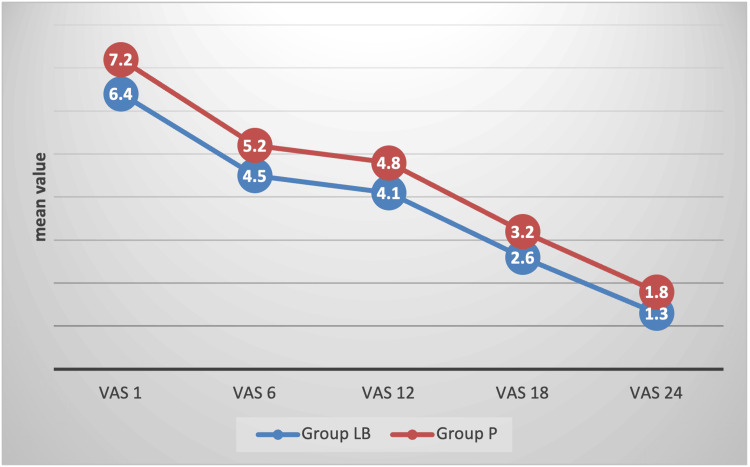
VAS score trend p<0.05 at all time points. VAS: Visual analog scale

In Group LB, the VAS scores showed a declining trend, starting at 6.4 ± 0.8 at one hour and dropping to 4.5 ± 1.5 at six hours, 4.1 ± 1.7 at 12 hours, 2.6 ± 1.3 at 18 hours, and 1.3 ± 0.6 at 24 hours. In Group P, the VAS scores also decreased. Statistically, VAS scores were significantly lower in Group LB as compared to Group P (Table 2, Figure 1).

**Table 2:** Pain scores assessed by VAS at follow-up VAS: Visual analog scale; * Independent t-test

VAS score	Group LB (n=40)	Group P (n=40)	p value
VAS 1	6.4 ± 0.8	7.2 ± 1.1	0.0004^*^
VAS 6	4.5 ± 1.5	5.2 ± 1.2	0.024^*^
VAS 12	4.1 ± 1.7	4.8 ± 1.1	0.032^*^
VAS 18	2.6 ± 1.3	3.2 ± 0.9	0.019^*^
VAS 24	1.3 ± 0.6	1.8 ± 0.5	0.0001^*^

In terms of the time to first rescue analgesia, compared to Group P, Group LB had significantly more time to first rescue analgesia (12 vs. five hours, p<0.0001).

In terms of the requirement of additional analgesia, compared to Group P, Group LB had significantly fewer patients with the requirement of additional analgesia (25 (62.5%) vs. 36 (90%), p=0.007). Even the total dose of fentanyl used for the patients in Group LB was significantly lower than the patients in Group P (3 ± 1 µg/kg vs. 6 ± 2 µg/kg, p<0.0001), as shown in Table 3.

**Table 3:** Additional analgesic requirement in first 24 hours ^‡ ^Fisher's exact test; ^# ^Independent t-test

Total analgesic requirement in first 24 hours	Group LB (n=40)	Group P (n=40)	p value
Required	25 (62.5%)	36 (90%)	0.007^‡ ^
Dose of fentanyl (µg/kg)	3 ± 1	6 ± 2	<0.0001^#^

**Figure 2:**
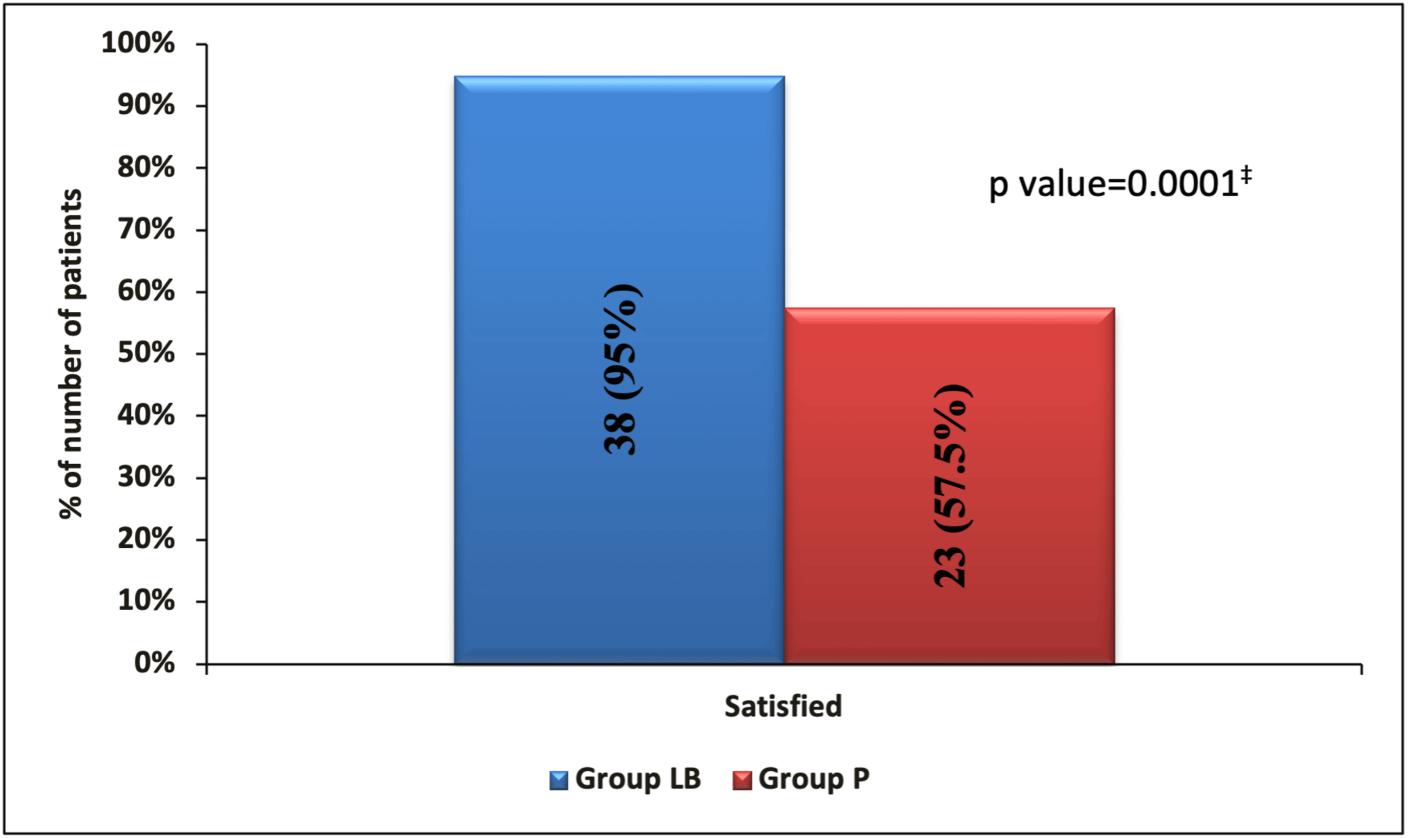
Satisfaction rate ^‡ ^Fisher's exact test

Compared to Group P, Group LB patients had lesser nausea (3 (7.5%) vs. 6 (15%), p=0.481) and vomiting (5 (12.5%) vs. 8 (20%), p=0.363) but were statistically comparable (Table 4). In terms of patient satisfaction, compared to Group P, Group LB had significantly higher patient satisfaction (38 (95%) vs. 23 (57.5%), p=0.0001) (Figure 2).

**Table 4:** Adverse effects ^† ^Chi-square test; ^‡ ^Fisher's exact test

Adverse effects	Group LB (n=40)	Group P (n=40)	p value
Nausea	3 (7.5%)	6 (15%)	0.481^‡^
Vomiting	5 (12.5%)	8 (20%)	0.363^†^

## Discussion

The current study is one of the landmark studies as it compares liver bed infiltration with levobupivacaine against intravenous paracetamol for managing pain after laparoscopic cholecystectomy. The liver bed infiltration technique was performed by administering levobupivacaine through the epigastric port from where the gallbladder was removed. The pertinent dose was selected since it is in the safe dose range of 7.5-15 mg. No previous published study has compared these two drugs. Our study found that intraperitoneal levobupivacaine provided adequate analgesia and reduced pain at the surgical site, and this reduction in pain was significantly more than intravenous analgesia using paracetamol. Even nausea and vomiting side effects were statistically comparable in both groups.

In comparison, previous research has mainly assessed the use of intraperitoneal bupivacaine, which was found to show consistent results in reducing pain at the surgical site, as well as shoulder pain [1]. Even a study by Khurana et al. [14] reported intraperitoneal bupivacaine provides adequate analgesia. However, one of the studies by Zmora et al. [15] found that intraperitoneal bupivacaine did not attenuate pain following laparoscopic cholecystectomy. This may be because, in their study, intraperitoneal bupivacaine was applied at the gallbladder bed following the dissection and removal of the gallbladder, which did not provide sufficient pain relief. In contrast, our study applied levobupivacaine infiltration at the liver bed. It is assumed that pain in cases of laparoscopic cholecystectomy may be generated by peritoneal irritation, especially in the right upper abdomen, and if a local anesthetic agent can be injected into the peritoneal cavity, it would significantly reduce the pain, which was observed in our study.

This also shows that the effect of bupivacaine needs to be differentiated from levobupivacaine since levobupivacaine has a longer duration of action and can block the sensory and motor nerve conduction at the local level by interacting with potassium channels and sodium channels, thus interfering with the pain transmission, which in cases of laparoscopic cholecystectomy is mainly incited by local irritation and pneumoperitoneum [16].

Furthermore, this was reflected in the reduction of demand for additional analgesia and total postoperative analgesia dose during the first 24 hours following laparoscopic cholecystectomy in our study. Such an effect has been consistently shown by levobupivacaine in various other studies of rhinoplasty, septoplasty, and mini-abdominoplasty [17,18]. 

Our study showed that the use of levobupivacaine for liver bed infiltration provided adequate pain relief, indicating that this method of intraperitoneal levobupivacaine holds superiority over intravenous analgesia with paracetamol and even intraperitoneal bupivacaine as used in the previous studies [16-18].

Moreover, since pain reduction was greater in Group LB, it allowed for better movement facilitation, improved respiration, and a return to normal functioning. Thus, overall patient satisfaction was also higher in Group LB as compared to Group P.

## Conclusions

Levobupivacaine used for liver bed infiltration provided adequate pain relief following laparoscopic cholecystectomy in comparison to paracetamol intravenous analgesia. Side effects like nausea and vomiting were statistically comparable in both groups. Overall, liver bed infiltration with levobupivacaine is a safe and effective option for controlling pain and increasing satisfaction among patients undergoing laparoscopic cholecystectomy.
